# Modeling of the Through-the-Thickness Electric Potentials of a Piezoelectric Bimorph Using the Spectral Element Method

**DOI:** 10.3390/s140203477

**Published:** 2014-02-20

**Authors:** Xingjian Dong, Zhike Peng, Hongxing Hua, Guang Meng

**Affiliations:** Institute of Vibration Shock & Noise, State Key Laboratory of Mechanical System and Vibration, Shanghai Jiao Tong University, Shanghai 200240, China; E-Mails: z.peng@sjtu.edu.cn (Z.P.); hhx@sjtu.edu.cn (H.H.); gmeng@sjtu.edu.cn (G.M.)

**Keywords:** spectral element method, piezoelectric bimorph, electric potential, sublayer, piecewise linear

## Abstract

An efficient spectral element (SE) with electric potential degrees of freedom (DOF) is proposed to investigate the static electromechanical responses of a piezoelectric bimorph for its actuator and sensor functions. A sublayer model based on the piecewise linear approximation for the electric potential is used to describe the nonlinear distribution of electric potential through the thickness of the piezoelectric layers. An equivalent single layer (ESL) model based on first-order shear deformation theory (FSDT) is used to describe the displacement field. The Legendre orthogonal polynomials of order 5 are used in the element interpolation functions. The validity and the capability of the present SE model for investigation of global and local responses of the piezoelectric bimorph are confirmed by comparing the present solutions with those obtained from coupled 3-D finite element (FE) analysis. It is shown that, without introducing any higher-order electric potential assumptions, the current method can accurately describe the distribution of the electric potential across the thickness even for a rather thick bimorph. It is revealed that the effect of electric potential is significant when the bimorph is used as sensor while the effect is insignificant when the bimorph is used as actuator, and therefore, the present study may provide a better understanding of the nonlinear induced electric potential for bimorph sensor and actuator.

## 1. Introduction

Piezoelectric materials generate electric potentials in response to mechanical stresses, and conversely, produce mechanical movements in response to electric potentials. Therefore, piezoelectric materials can be used both as actuators and sensors, they transform electrical energy into mechanical energy, and *vice versa*. To achieve practically meaningful actuation and sensing capabilities, a piezoelectric bimorph consisting of two piezoelectric layers is widely used [1,2]. A broad range of electromechanical applications have been reported, such as electroacoustic transducers [3,4], medical devices [5], microcantilever biosensors [6], and atomic force microscope (AFM) cantilevers [7]. However, before piezoelectric bimorphs can be utilized in all these applications, it is first necessary to investigate both the global responses and the local responses, e.g., the deflection and the distribution of the electric potential across the thickness.

There have been many theories and models developed for analyzing piezoelectric bimorph structures with emphasis on approximating the mechanical displacement and electric potential. By carrying out exact 3-D analytical solutions for the simply supported piezoelectric plate [8,9], it is shown that the distribution of the electric potential across the thickness is nearly quadratic. This implies that the assumption of linear distribution of the electric potential across the thickness adopted by many numerical models [10,11] cannot address this nonlinear electric potential. Since exact 3-D analytical solutions are not available for more general cases of loading and boundary conditions, the introduction of the finite element (FE) method is desirable. A considerable amount of literature has been published on the FE analysis of piezoelectric smart structures [12–14]. Among these works, the simplest and often used model is the equivalent single layer (ESL) model in which the displacement and strain functions are assumed to be continuous through the thickness. There are two main kinds of theories used for ESL models. One is the classical laminated plate theory (CLPT) [15,16], and the other one is the shear deformation theory, which branches out into first-order shear deformation theory (FSDT) [17,18] and higher order shear deformation theory (HSDT) [19,20]. The ESL model is simple and capable of predicting the global responses of the bimorph, but it does not account for the nonlinear distribution of the electric potential across the thickness. To overcome this shortcoming, the FE model using the layer-wise theory [21–24] or the sublayer theory [2,25–28] has been recommended. In the latter case, the piezoelectric layer is divided into appropriate number of thin sublayers. For each of these sublayers, a linear electric potential distribution across the plate thickness is assumed. It is further expected that the quadratic distribution of the electric potential across the plate thickness can be accurately approached with more sublayers adopted.

Generally, accurately simulation of the local responses of the piezoelectric bimorph structures would inevitably lead to a very dense FE mesh when using the FE method. Hence, conventional FE simulation becomes computationally very inefficient. A more efficient method is the spectral element (SE) method which combines the geometric flexibility of FE method with the high accuracy of the pseudo spectral method. This method was first presented by Patera in the mid 1980s [29]. In fact, the SE method and FE method are closely related and built on the same ideas. The main difference between them is that SE method uses orthogonal polynomials, such as Legendre and Cheybysev polynomials, in the shape functions. The SE method results naturally in diagonal mass matrices which is a distinct advantage over traditional FE method especially for transient analysis. Moreover, to have an accurate simulation with the conventional FE method, a mesh with a large number of elements and degrees of freedom (DOFs) is inevitably needed. The SE method, in which the polynomial order is increased and the mesh size is decreased, can be used to overcome this problem. The SE method has been widely applied to many engineering problems related to acoustics, fluid dynamics and seismology [30–35]. Recently, the SE method has been extensively used to investigate the wave propagation problems for the purpose of damage detection in structures [36,37]. However, according to the authors' best knowledge, the SE method has not been previously used for accurately modeling of the through-the-thickness electric potentials for piezoelectric bimorphs.

For the purpose of accurately representing the mechanical displacement and the electric potential, a reasonable choice is to use the ESL model for the mechanical variables and the layer-wise theory or the sublayer theory for the electric variables. In the present work, we attempt to combine the merits of the SE method and the sublayer model. More specifically, the mechanical variables, *i.e.*, the displacements, are described based on FSDT. The electrical variables, *i.e.*, the potentials, are described using the sublayer model. SE method is then utilized to deduce the governing equations. Legendre orthogonal polynomials are adopted in the interpolation function to improve the accuracy. To validate the effectiveness and the capability of the present model, numerical simulations for a simply supported piezoelectric bimorph with two different load cases, *i.e.*, a uniform pressure load applied to the top surface and a uniform potential applied to the top and bottom surfaces, are carried out. The results obtained by the present approach are then compared to those coming from the coupled 3-D FE simulations using ABAQUS. The comparisons show the good accuracy and efficiency of SE method for modeling of the through-the-thickness electric potentials of the piezoelectric bimorph.

## 2. Mathematical Formulation

### 2.1. Constitutive Relationships, Displacement and Strain

**Figure 1.**
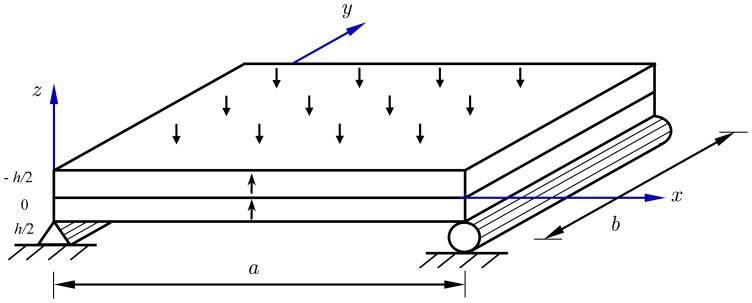
Geometry of a piezoelectric bimorph.

A piezoelectric bimorph made of two identical PZT-4 piezoelectric layers, which has been investigated by Fernandes [1], is considered here. The PZT-4 layer is assumed to behave in a linear orthotropic manner with small displacements and strains. As depicted in Figure 1, both piezoelectric layers have the same thickness 0.5 h and are poled in the same direction. The *x*-*y* plane of the coordinate system *x*-*y*-*z* coincides with the middle plane of the bimorph, and the *z* axis is defined normal to the middle plane following the right-hand rule. This work aims to investigate the problem of a simply supported piezoelectric bimorph under a uniform pressure load or an applied electric potential in the framework of linear theory of piezoelectricity. Assuming the PZT-4 layers work under isothermal conditions, the pyroelectric effects and thermomechanical couplings are not taken into account. Consequently, a linear constitutive relationship addressing both the direct and converse piezoelectric effects is utilized for the analysis of the piezoelectric bimorph, which can be written as:

(1) $$\begin{array}{c} \text{σ}=\text{c}\varepsilon -{\text{e}}^{\text{T}}\text{E}\\ \text{D}=\text{e}\varepsilon +\text{gE} \end{array}$$

where **σ** = [*σ_x_ σ_y_ σ_z_ τ_yz_ τ_zx_ τ_xy_*]^T^ and **ε** = [*ε_x_ ε_y_ ε_z_ γ_yz_ γ_zx_ γ_xy_*]^T^ represent stress vector and strain vector, respectively. **E** = [*E_x_ E_y_ E_z_*]^T^, the electric field vector, **D** = [*D_x_ D _y_ D _z_*]^T^, the electric displacement vector, **c**, the elastic coefficient matrix, **g**, the dielectric coefficient matrix, and **e**, the piezoelectric stress coefficient matrix.

An ESL model adopting the FSDT is adopted to describe the mechanical displacement. The displacement field of a piezoelectric bimorph based on FSDT takes on the form [17,18]:

(2) $$\begin{array}{l} u\;x,y,z,t=\bar{u}(x,y,t)+z\bar{\alpha }(x,y,t)\\ \upsilon \;x,y,z,t=\bar{v}(x,y,t)+z\bar{\beta }(x,y,t)\\ w\;x,y,z,t=\bar{w}(x,y,t) \end{array}$$

where *u̅*, *υ̅*, *w̅* denote the displacements of an arbitrary point on the mid plane *z* = 0, *α̅* and −*β̅* denote the rotations of a transverse normal about the *y* and *x* axes, respectively. In the FSDT, the transverse shear strains are assumed to be constant with respect to the thickness coordinate. The constant state of transverse shear strains across the thickness is a gross approximation of the true strain field, which is at least quadratic through the thickness.

We define:

(3) $$\text{U}={\left[\begin{array}{ccc} u & \upsilon & w \end{array}\right]}^{\text{T}}$$

(4) $$\text{U}={\left[\begin{array}{lllll} \bar{u} & \bar{\upsilon } & \bar{w} & \bar{\alpha } & \bar{\beta } \end{array}\right]}^{T}$$

where **U** is the displacement vector, and **U̅** is a generalized displacement vector. Then Equation (2) can be written in matrix form as:

(5) $$\text{U}=\text{Z}\bar{\text{U}}$$

where:

(6) $$\text{z}=\left[\begin{array}{lllll} 1 & 0 & 0 & z & 0\\ 0 & 1 & 0 & 0 & z\\ 0 & 0 & 1 & 0 & 0 \end{array}\right]$$

The infinitesimal strain components associated with the displacements are given by:

(7) ε=LU

where **L** is the derivation operator defined as:

(8) $$\text{L}={\left[\begin{array}{llllll} \frac{\partial }{\partial x} & 0 & 0 & 0 & \frac{\partial }{\partial z} & \frac{\partial }{\partial y}\\ 0 & \frac{\partial }{\partial y} & 0 & \frac{\partial }{\partial z} & 0 & \frac{\partial }{\partial x}\\ 0 & 0 & \frac{\partial }{\partial z} & \frac{\partial }{\partial y} & \frac{\partial }{\partial x} & 0 \end{array}\right]}^{T}$$

### 2.2. Approximations for Displacements

**Figure 2.**
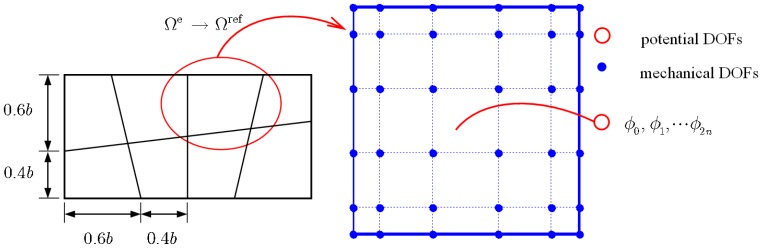
Discretization of a plate and an example of spectral element.

The Legendre polynomials based SE method can be described as follows: the bimorph is firstly discretized using a set of non-overlapping rectangular elements, as in the traditional FE method. Each rectangular element, denoted by Ω^e^, is then mapped to a reference element, denoted by Ω^ref^: *ξ* ∈ [−1,1]× *η* ∈ [−1,1], using an invertible local mapping. The discretization procedure is illustrated in Figure 2.

Subsequently, a set of nodes, denoted by *ξ_i_*, *η_j_* are defined in the local coordinate system *ξ*−*η* of the reference element Ω^ref^ as roots of the following polynomial expression:

(9) $$\{\begin{array}{c} (1-\xi^{2})P_{N}^{\prime }(\xi )=0\\ (1-\eta^{2})P_{N}^{\prime }(\eta )=0 \end{array}$$

where *P_N_* is the *N*-th order Legendre polynomial. In fact, the nodes are the 2-D Gauss-Lobatto-Legendre (GLL) points. In contrast to the classical FE method, the distribution of nodes is irregular, as shown in Figure 2. In the current formulation, the 5-th order Legendre polynomial is chosen, hence 36 nodes can be specified in the reference element Ω^ref^, as depicted in Figure 2.

The 1-D shape functions at the 1-D GLL points *ξ_i_* are defined as [36]:

(10) $$\begin{array}{cc} h_{i}(\xi )=\frac{-1}{N(N+1)P_{N}(\xi_{i})}\frac{\left(1-\xi^{2})P_{N}^{\prime }(\xi \right)}{\xi -\xi_{i}} & \text{for}\;i=1,\cdots ,N+1 \end{array}$$

An important property of these interpolation functions is the discrete orthogonality expressed as:

(11) $$h_{i}(\xi_{j})=\delta_{ij}$$

where *δ_ij_* denotes the Kronecker delta. The 2-D shape functions are constructed as a tensor product of the 1-D ones:

(12) $$\begin{array}{cc} \Psi_{ij}(\xi ,\eta )=h_{i}(\xi )h_{j}(\eta ) & \text{for}\;i,j=1,\cdots ,N+1 \end{array}$$

**Figure 3.**
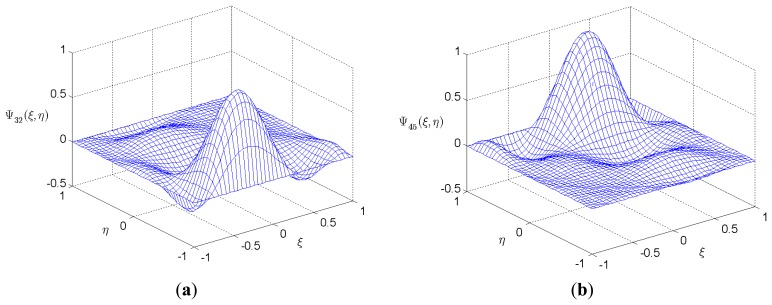
Selected shape functions for a 36-node spectral element. (**a**) Ψ_32_(*ξ*,*η*); (**b**) Ψ_45_(*ξ*,*η*).

Figure 3 shows two examples of the 2-D shape functions which indicate that each shape function has the value 1 at one node and vanish at all other nodes.

Coordinates *x* and *y* within each Ω^e^ may be uniquely related to *ξ* and *η* upon the invertible mapping:

(13) $$\langle x(\xi ,\eta ),y(\xi ,\eta )\rangle =\overset{6}{\underset{i=1}{\text{∑}}}\overset{6}{\underset{j=1}{\text{∑}}}\Psi_{ij}(\xi ,\eta )\langle x_{ij},y_{ij}\rangle$$

where *x_ij_* and *y_ij_* denote the coordinates of *x* and *y*, respectively, of the element nodes *ξ_i_*, *η_j_*. The generalized displacements *u̅*, *υ̅*, *w̅*, *α̅* and *β̅* over an reference element Ω^ref^ are discretized by the 2-D shape functions as:

(14) $$\langle \bar{u}(\xi ,\eta ),\bar{\upsilon }(\xi ,\eta ),\bar{w}(\xi ,\eta ),\bar{\alpha }(\xi ,\eta ),\bar{\beta }(\xi ,\eta )\rangle =\overset{6}{\underset{i=1}{\text{∑}}}\overset{6}{\underset{j=1}{\text{∑}}}\Psi_{ij}(\xi ,\eta )\langle {\bar{u}}_{ij},{\bar{\upsilon }}_{ij},w_{ij}\rangle$$

where *u̅_ij_*, *υ̅_ij_*, *w̅_ij_*, *α̅_ij_* and *β̅_ij_* are the nodal values of the generalized displacements. The discrete element nodal displacement vector is expressed as:

(15) $${\text{q}}^{\text{e}}={\left[\begin{array}{llll} {\text{q}}_{11}^{\text{T}} & {\text{q}}_{12}^{\text{T}} & \cdots & {\text{q}}_{66}^{\text{T}} \end{array}\right]}^{\text{T}}$$

where **q**_*ij*_ is the displacement vector of the node *ξ_i_*, *η_j_*:

(16) $${\text{q}}_{ij}={\left[\begin{array}{lllll} {\bar{u}}_{ij} & {\bar{\upsilon }}_{ij} & {\bar{w}}_{ij} & {\bar{\alpha }}_{ij} & {\bar{\beta }}_{ij} \end{array}\right]}^{\text{T}}$$

Substituting Equations (14) into Equation (15) yields:

(17) $$\text{U}={\text{N}}_{u}{\text{q}}^{\text{e}}$$

where **N***_u_* is the displacement shape function matrix which can be expressed as:

(18) $${\text{N}}_{u}=\text{Z}\left[\begin{array}{llll} {\text{N}}_{11} & {\text{N}}_{12} & \cdots & {\text{N}}_{66} \end{array}\right]$$

with:

(19) $${\text{N}}_{ij}=\Psi_{ij}{\text{I}}_{5\times 5}$$

where **I**_5×5_ is a 5×5 identity matrix. Substituting Equation (17) into Equation (7) yields:

(20) $$\varepsilon ={\text{B}}_{u}{\text{q}}^{\text{e}}$$

where **B***_u_* is strain-displacement matrix which can be written as:

(21) $${\text{B}}_{u}=\text{L}{\text{N}}_{u}$$

### 2.3. Approximations for Electric Potential

**Figure 4.**
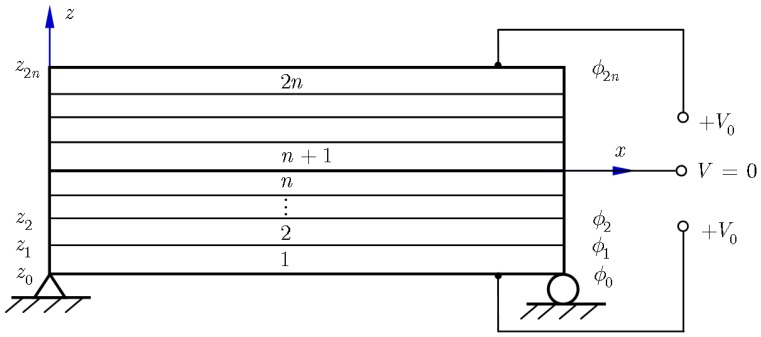
A sublayer model for a piezoelectric bimorph.

For the purpose of accurately modeling the distribution of the electric potential across thickness, each layer of the piezoelectric bimorph is subdivided mathematically into *n* thinner sublayers. As shown in Figure 4, the sublayers are numbered in top-to-bottom order. The *z* coordinates of the top and bottom surfaces of the *i*-th sublayer are denoted by *z_i_* and *z_i_*_−1_, respectively. In each sublayer, the distribution of the electric potential *ϕ^i^*(*z*) is assumed to be linear across the thickness such that:

(22) $$\phi^{i}(z)={\text{N}}_{\phi }^{i}{\tilde{\Phi }}^{i}$$

where ${\text{N}}_{\phi }^{i}$ is the interpolation function and **Φ̃***^i^* is a column matrix composed of the electric potentials at the top and the bottom surfaces of the *i*–th sublayer, which can be expressed as:

(23) $$\begin{array}{ccc} {\text{N}}_{\phi }^{i}=\frac{1}{h_{i}}[\begin{array}{cc} z_{i}-z & z-z_{i-1} \end{array}], & h_{i}=z_{i}-z_{i-1}, & z_{i-1}\leq z\leq z_{i} \end{array}$$

(24) $${\tilde{\Phi }}^{i}={\left[\begin{array}{cc} \phi_{i-1} & \phi_{i} \end{array}\right]}^{T}$$

In this way, the assumption of linear distribution of electric potential across the thickness is used not in the whole piezoelectric layer, but in each sublayer instead. As a result, the electric potential is approximated as piecewise linear across the thickness and it is expected that the quadratic distribution of the electric potential across the bimorph thickness can be approached with more sublayers adopted. As mentioned before, the mechanical displacement field is approximated using ESL model based on FSDT and the piezoelectric bimorph is discretized using 2-D mesh. To keep the compatibility, each sublayer of the piezoelectric layer is also discretized using the same mesh. Consequently, an element potential vector **Φ**^e^ is then introduced in the spectral plate finite element, which is defined as:

(25) $$\Phi^{\text{e}}={\left[\begin{array}{llll} \phi_{0} & \phi_{1} & \cdots & \phi_{2n} \end{array}\right]}^{\text{T}}$$

The surface potential of the sublayer, *ϕ_i_*, is assumed to be constant over the element and *ϕ*_0_, *ϕ*_1_, ⋯ *ϕ*_2_*_n_* are treated as elemental DOFs, as illustrated in Figure 2. Furthermore, the top and bottom surfaces of the piezoelectric layers are always coated with metallic coatings of zero thickness and the potentials on the electrodes should be taken as independent of *x*, *y*. Thus the present method combines an ESL theory for the displacement and a piecewise linear approximation for the electric potential. Under the quasi-electrostatic approximation, the electric field and the electric potential in each sublayer have the following relationship:

(26) $${\text{E}}^{i}(z)=-{\text{B}}_{\phi }^{i}{\tilde{\Phi }}^{i}$$

where **E***^i^*(*z*) is the electric field of the *i*-th sublayer, ${\text{B}}_{\phi }^{i}$ is the electric field-potential matrix, given by:

(27) $${\text{B}}_{\phi }^{i}=\nabla {\text{N}}_{\phi }^{i}$$

#### 2.4. Governing Equations

By applying Hamilton's principle, the elementary dynamic equations for the piezoelectric bimorph plate can be obtained:

(28) $$\begin{array}{r} {\text{M}}_{uu}^{\text{e}}{\ddot{\text{q}}}^{\text{e}}+{\text{K}}_{uu}^{\text{e}}{\text{q}}^{\text{e}}+{\text{K}}_{u\phi }^{\text{e}}\Phi^{\text{e}}={\text{F}}_{u}^{\text{e}}\\ {\text{K}}_{\phi u}^{\text{e}}{\text{q}}^{\text{e}}+{\text{K}}_{\phi \phi }^{\text{e}}\Phi^{\text{e}}={\text{F}}_{\phi }^{\text{e}} \end{array}$$

where ${\text{M}}_{uu}^{\text{e}}$ denotes the element mass matrix; ${\text{K}}_{uu}^{\text{e}}$, mechanical stiffness matrix; ${\text{K}}_{u\phi }^{\text{e}}$ and ${\text{K}}_{\phi u}^{\text{e}}$ the piezoelectric coupling matrices; ${\text{K}}_{\phi \phi }^{\text{e}}$ the dielectric permittivity matrix; ${\text{F}}_{u}^{\text{e}}$ the vector of externally applied force; and ${\text{F}}_{\phi }^{\text{e}}$ the vector of externally applied charge, respectively:

(29) $${\text{M}}_{uu}^{\text{e}}=\overset{2n}{\underset{i=1}{\text{∑}}}h_{i}\int_{-1}^{1}\int_{-1}^{1}\rho {\text{N}}_{u}^{\text{T}}{\text{N}}_{u}\left|\text{J}\right|\text{d}\xi \text{d}\eta$$

(30) $${\text{K}}_{uu}^{\text{e}}=\overset{2n}{\underset{i=1}{\text{∑}}}h_{i}\int_{-1}^{1}\int_{-1}^{1}{\text{B}}_{u}^{\text{T}}{\text{cB}}_{u}\left|\text{J}\right|\text{d}\xi \text{d}\eta$$

(31) $${\text{K}}_{u\phi }^{\text{e}}={\left[{\text{K}}_{\phi u}^{\text{e}}\right]}^{\text{T}}=\overset{2n}{\underset{i=1}{\text{∑}}}h_{i}\int_{-1}^{1}\int_{-1}^{1}{\text{B}}_{u}^{\text{T}}{\text{e}}^{\text{T}}{\text{B}}_{\phi }^{i}\left|\text{J}\right|\text{d}\xi \text{d}\eta$$

(32) Kϕϕe=−∑i=12nhi∫−11∫−11BϕigTBϕi|J|dξdη

(33) $${\text{F}}_{u}^{\text{e}}=\int_{-1}^{1}\int_{-1}^{1}{\text{N}}_{u}^{\text{T}}{\text{P}}_{s}\left|\text{J}\right|\text{d}\xi \text{d}\eta$$

(34) $${\text{F}}_{\phi }^{\text{e}}={\underset{i}{\text{∑}}\int_{-1}^{1}\int_{-1}^{1}-{\text{N}}_{\phi }^{i}}^{\text{T}}{\text{q}}_{s}\left|\text{J}\right|\text{d}\xi \text{d}\eta$$

where *ρ* is the mass density, **P***_s_* is the surface force vector, **q***_s_* is the surface charge density vector. **J** is the well-known Jocobian matrix of the mapping (13) which is defined as:

(35) $$\text{J}=\left[\frac{\partial (x,y)}{\partial (\xi ,\eta )}\right]=\left[\begin{array}{cc} \frac{\partial x}{\partial \xi } & \frac{\partial y}{\partial \xi }\\ \frac{\partial x}{\partial \eta } & \frac{\partial y}{\partial \eta } \end{array}\right]$$

The GLL integration rule is then used to calculate the characteristic matrices and the nodal force vector in Equation (28) at the elemental level [36]. In this study, the interface between the two PZT layers is grounded. Two sets of electric boundary conditions are considered, *i.e.*, (1) sensor function with the top and bottom surfaces grounded and a uniform pressure load of *S*_0_ = 1,000N/m^2^ applied to the upper surface, and Equation (2) actuator function with an electric potential of *V*_0_ = 50V applied to the top and bottom surface of the bimorph. By applying the electric boundary conditions, the DOFs for the electric potential are condensed out such that Equation (28) is finally of the form:

(36) $${\text{M}}_{uu}^{\text{e}}{\ddot{\text{q}}}^{\text{e}}+{\text{K}}_{uu}^{\text{e}}+{\text{K}}_{p}^{\text{e}}\;{\text{q}}^{\text{e}}={\text{F}}_{u}^{\text{e}}+{\text{F}}_{a}^{\text{e}}$$

where ${\text{K}}_{p}^{\text{e}}$ denotes the mechanical stiffness matrix induced by the electromechanical coupling of PZT-4 layer, and ${\text{F}}_{a}^{\text{e}}$ denotes the mechanical forces induced by the applied voltages of piezoelectric actuators [2]. The electric potential is then recovered by the inverse process of the aforementioned condensation. Assembling all elementary equations, one can have a global dynamic system equation:

(37) $${\text{M}}_{uu}\ddot{\text{q}}+{\text{K}}_{uu}+{\text{K}}_{p}\;\text{q}={\text{F}}_{u}+{\text{F}}_{a}$$

where **M***_uu_*, **K***_uu_*, **K***_p_*, **F***_u_* and **F***_a_* are the assembled counterparts of matrices ${\text{M}}_{uu}^{\text{e}}$, ${\text{K}}_{uu}^{\text{e}}$, ${\text{K}}_{p}^{\text{e}}$, ${\text{F}}_{u}^{\text{e}}$ and ${\text{F}}_{a}^{\text{e}}$; **q** is the global nodal displacement vector. Since the DOFs for the sublayer electric potentials have been condensed out, this approach will not result in a large number of potential DOFs. For the purpose of static analysis, the governing equations in Equation (37) reduces to:

(38) $${\text{K}}_{uu}+{\text{K}}_{p}\;\text{q}={\text{F}}_{u}+{\text{F}}_{a}$$

## 3. Numerical Results

In this section, the derived SE model is converted into a numerical code and case studies are carried out to validate the effectiveness and the capability of the present model for predicting both the global responses and the local responses, *i.e.*, the deflections of the bimorph and the distribution of the electric potential across the bimorph thickness. A simply supported rectangular piezoelectric bimorph shown in Figure 1, which has been investigated by Fernandes [1], is considered here. The material constants of PZT-4 are given as:

(39) $$\text{c}=\left[\begin{array}{llllll} 139 & 77.8 & 74.3 & 0 & 0 & 0\\ 77.8 & 139 & 74.3 & 0 & 0 & 0\\ 74.3 & 74.3 & 115 & 0 & 0 & 0\\ 0 & 0 & 0 & 25.6 & 0 & 0\\ 0 & 0 & 0 & 0 & 25.6 & 0\\ 0 & 0 & 0 & 0 & 0 & 30.6 \end{array}\right]\text{GPa}$$

(40) $$\text{e}=\left[\begin{array}{llllll} 0 & 0 & 0 & 0 & 12.7 & 0\\ 0 & 0 & 0 & 12.7 & 0 & 0\\ -5.2 & -5.2 & 15.1 & 0 & 0 & 0 \end{array}\right]\;\text{C}/{\text{m}}^{2}$$

(41) $$\text{g}=\left[\begin{array}{lll} 13.06 & 0 & 0\\ 0 & 13.06 & 0\\ 0 & 0 & 11.51 \end{array}\right]\;\text{nF}/\text{m}$$

The length *a* and width *b* of the bimorph are 25 mm and 12.5 mm respectively. Two values of slenderness ratio, *S* = *a*/*h* = 5 and, *S* = 50 which represent the thick and thin bimorph plate, respectively, are considered. Unless otherwise stated, the order of Legendre polynomial is chosen as 5, and the mesh in Figure 3 is used in this work. Two load cases corresponding respectively to sensor function and actuator function are considered. To overcome the ill condition problem resulted from the huge difference of the element values of ${\text{K}}_{uu}^{\text{e}}$ and ${\text{K}}_{\phi \phi }^{\text{e}}$ in magnitudes, Equation (28) is rewritten using dimensionless variables. Consequently, the numerical results for the deflection and the electric potential are given in dimensionless units as:

(42) $$W,\Phi =\frac{c_{11}}{hS_{0}}(w,\phi /E_{0})\;\text{for sensor function}$$

(43) $$W,\Phi =\frac{E_{0}}{V_{0}}(w,\phi /E_{0})\;\text{for actuator function}$$

where the amplification factor *E*_0_ is taken as *E*_0_ = 10^10^V/m. For the purpose of comparison, a coupled 3-D analysis is carried out using 20-noded hexahedral 3-D piezoelectric elements (C3D20RE) with a mesh size of 40×20×10 in ABAQUS and the results from the coupled 3-D FE analysis are taken as accurate.

### 3.1. Sensor Function

**Figure 5.**
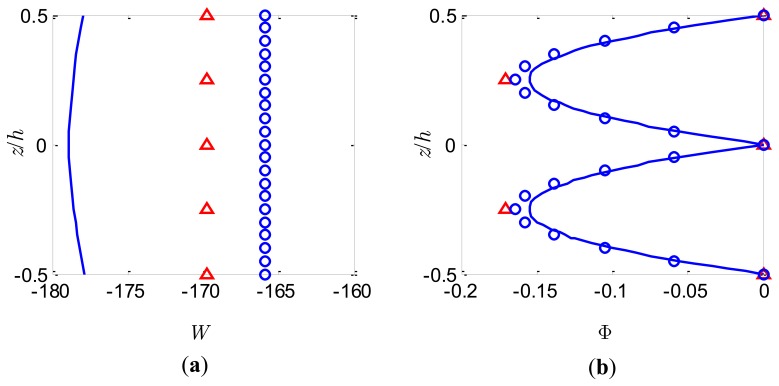
Bimorph sensor of *S* = 5 under pressure load. (**a**) Dimensionless deflection; (**b**) Dimensionless electric potential. 3-D FE analysis (full line), present model with *n* = 5 (triangles) and present model with *n* = 5 (small circles).

**Figure 6.**
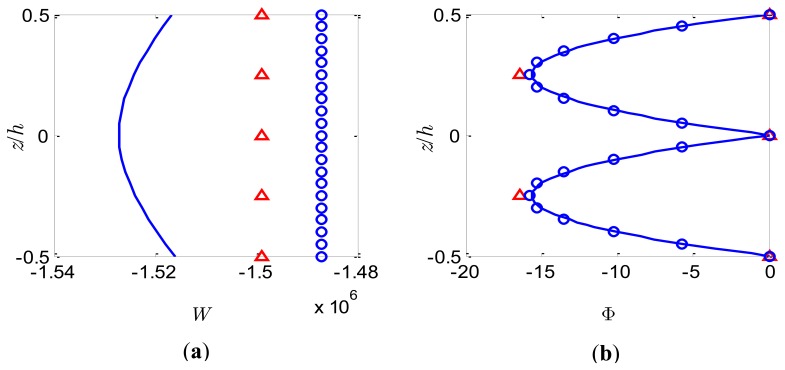
Bimorph sensor of *S* = 50 under pressure load. (**a**) Dimensionless deflection; (**b**) Dimensionless electric potential. 3-D FE analysis (full line), present model with *n* = 2 (triangles) and present model with *n* = 10 (small circles).

For this case a uniform pressure load of *S*_0_ = 1,000N/m^2^ is applied to the upper surface and the bimorph is used as a sensor with the top and bottom surfaces grounded. The variations of both the deflection *W* and the electric potential Φ across the bimorph thickness at the centre of the bimorph plate (*x* = 0.5*a*, *y* = 0.5*b*) for the slenderness ratio *S* = 5 and *S* = 50 are shown in Figures 5 and 6, respectively. It can be observed from Figure 5a and Figure 6a that the deflection *W* estimated by the present method adopting different number of sublayers is constant through the thickness and it is a good approximation of the nonlinear distribution described by the coupled 3-D analysis. The present model based on FSDT with assumption of uniform deflection through the thickness cannot predict the nonlinear variation of *W* through the thickness. The electric potentials induced by the deformation of the bimorph through the direct piezoelectric effects are shown in Figures 5b and 6b. It is observed that the distribution of the electric potential Φ across the thickness provided by the present approach with more than 2 sublayers is in good agreement with the nonlinear distribution predicted by the coupled 3-D analysis. Furthermore, it is expected that with more sublayers adopted the quadratic distribution of the electric potential Φ across the bimorph thickness can be accurately approached without introducing any higher-order electric potential assumptions. However, the conventional linear electric potential assumption [38] will result in an inaccurate prediction of the local electric potential response for the case of sensors. The curves in Figures 5 and 6 are symmetrical with respect to the interface between the two PZT layers. It should be highlighted that although the present method cannot predict accurately the distribution of *W* across the bimorph thickness, it may be able to provide good approximate results for Φ with appropriate number of sublayers for both thick and thin bimorph plates.

### 3.2. Actuator Function

**Figure 7.**
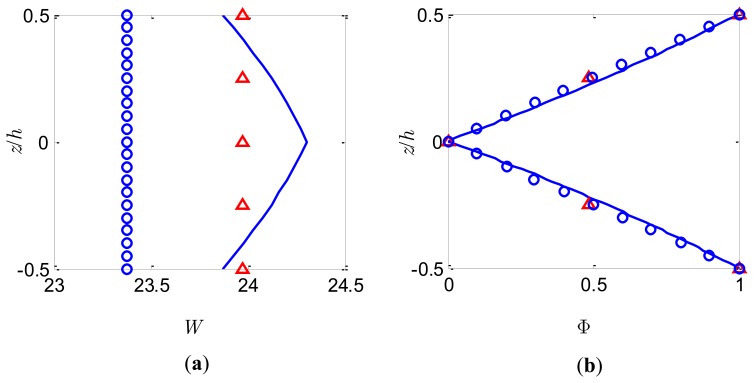
Bimorph actuator of *S* = 5 under potential load. (**a**) Dimensionless deflection; (**b**) Dimensionless electric potential. 3-D FE analysis (full line), present model with *n* = 2 (triangles) and present model with *n* = 10 (small circles).

**Figure 8.**
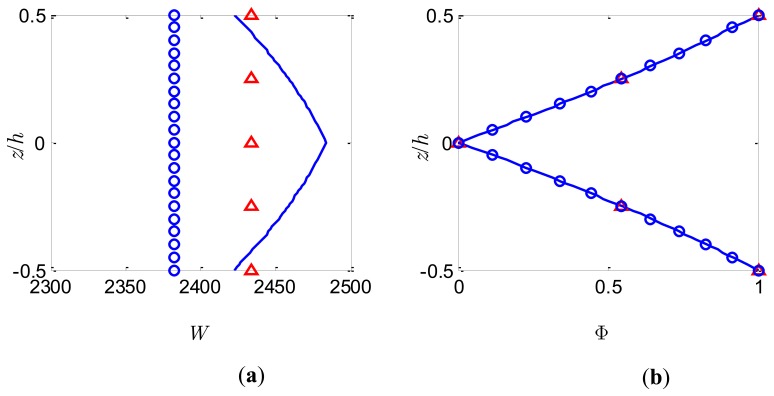
Bimorph actuator of *S* = 50 under potential load. (**a**) Dimensionless deflection; (**b**) Dimensionless electric potential. 3-D FE analysis (full line), present model with *n* = 2 (triangles) and present model with *n* = 10 (small circles).

To achieve practically meaningful actuation capabilities and guarantee that the piezoelectric material behaves linearly, an electric potential of *V*_0_ = 50V is applied to the top and bottom surfaces of the bimorph with intermediate electrode grounded. The through-the-thickness variations of *W* and Φ at the centre of the plate for *S* = 5 and *S* = 50 are shown in Figures 7 and 8, respectively.

Once again, the present model based on FSDT cannot predict accurately the through-the-thickness distribution of *W*. Similar to the previous observation, the constant deflection *W* through the thickness calculated by the present method adopting different number of sublayers is a good approximation of the nonlinear distribution provided by the coupled 3-D analysis. It is noticed that as the sublayer number increases a smaller deflection is obtained which is also pointed out by Wang [2]. The electric potentials at the centre of the plate are plotted in Figure 7b and Figure 8b for the slenderness ratio *S* = 5 and *S* = 50, respectively. It can be observed that the almost linear distribution of Φ across the thickness predicted by the present method for both thick and thin bimorph plates is in excellent agreement with the coupled 3-D analysis, indicating that the nonlinear induced electric potential is insignificant compared to the externally applied potential. Consequently, the conventional linear electric potential assumption [38] may be accurate enough to calculate the local electric potential response for the case of actuators.

## 4. Conclusions

The present work aims to develop an efficient SE model with electric potential DOFs for the static electromechanical response of a piezoelectric bimorph. The approach is the combination of an ESL model based on FSDT for the mechanical displacement with a sublayer model based on the piecewise linear approximation for the electric potential. 2-D GLL shape functions are used to discretize the displacements and then the governing equation of motion is derived following the standard SEM procedure. By applying the electric boundary conditions, the DOFs for the electric potential are condensed out such that the present model will not result in a large number of potential DOFs.

Numerical simulations based on the present model are carried out for two different load cases, *i.e.*, a uniform pressure load applied to the top surface and a uniform potential applied to the top and bottom surfaces. To validate the effectiveness and the capability of the present model for investigation of both global and local response of the piezoelectric bimorph, the numerical results thus obtained are compared to those from 3-D analysis using ABAQUS. The results indicate that the deflection *W* estimated by the present method is a good approximation of the nonlinear distribution predicted by the coupled 3-D analysis. It is further shown that the present model provides very accurate prediction for the electric potential distributions across the bimorph thickness even for rather thick bimorph plate without introducing any higher-order electric potential assumptions. It is also revealed that the conventional linear electric potential model is accurate enough to predict the local electric potential response for the case of actuators. This observation consists with the previous findings proposed by Yang [39]. One of the limitations is that the deflection *W* across the thickness is constant. Nevertheless, it is accurate enough to investigate the global response of the piezoelectric bimorph. The present work is important for researchers to better understand the nonlinear induced electric potential for bimorph sensor and actuator. An important extension of the present research is to study the vibration characteristics of the piezoelectric bimorph based on SE method. The influence of the induced stiffness matrix on the natural frequencies of the bimorph plate under various electric boundary conditions is to be investigated. The convergence study of the present model with respect to the order of the Legendre polynomial is also a practical and interesting problem to be conducted.
